# Comparative Plastome Analysis of *Artocarpus* Species in China: Insight Into Adaptive Evolution and Mutational Hotspot Regions

**DOI:** 10.1002/ece3.72881

**Published:** 2026-01-02

**Authors:** Ru‐Li Zhang, Xian‐Huang Li, Shu‐Mei Nuo, Bi‐Lin Li, Ming‐Song Peng, Wei‐ying Li, Yun Zhou, Dong Yan, Zhang‐Hong Dong

**Affiliations:** ^1^ Southwest Forestry University Kunming China; ^2^ Forestry Bureau of Nayong County Nayong China; ^3^ Forestry Research Institute of Meizhou Academy of Agriculture and Forestry Sciences Meizhou China; ^4^ Guangdong Engineering Technology Research Center (Meizhou) for the Development and Use of the Forest Characteristic Resources Meizhou China; ^5^ Lushui Bureau of Gaoligong Mountains National Nature Reserve Lushui China; ^6^ Forestry and Grassland Technique Extention Station of Baoshan City Baoshan China; ^7^ Nujiang Management Bureau, Gaoligongshan National Nature Reserve Lushui China

**Keywords:** *Artocarpus*, highly variable regions, phylogenetic relationship, plastomes, selective pressure

## Abstract

The genus *Artocarpus* J. R. Forst. & G. Forst., with about 70 species worldwide, contains roughly 15 species in China. Nevertheless, the phylogenetic relationships among these Chinese *Artocarpus* are incompletely understood. Here, we analyzed plastomes of ten Chinese *Artocarpus* species. These plastomes have a typical quadripartite structure, and sizes ranging from 160,184 bp to 161,009 bp. Simple sequence repeats (SSRs) numbered 73 to 95, while dispersed repeats ranged from 51 to 81. The protein‐coding genes displayed comparable RSCU patterns with minor variations. The genes ycf1 and ndhF showed varying degrees of expansion and contraction at their boundaries. Analysis of selective pressures in 72 protein‐coding genes revealed a predominance of purifying selection, with an average Ka/Ks ratio of 0.23, as well as evidence of positive selection in specific genes. Positive selection was detected in the genes *atp*F, *rpo*C2, *rpo*C1, *cem*A, *rpl*20, *clp*P, *pet*D, *rps*8, *rps*3, *rps*19, *ycf*2, *ycf*1, and *ndh*D within the plastomes of ten *Artocarpus* species in China. Phylogenetic analyses demonstrated a high degree of consistency in their main topological structures, dividing the genus *Artocarpus* into Clade A and Clade B. The highly variable regions *trn*H‐*psb*A, *trn*G‐UCC‐*trn*R‐UCU, *trn*S‐UGA, *trn*T‐UGU‐*trn*L‐UAA, and *rps*19 were suggested as molecular markers for distinguishing between these clades. Additionally, the study also identified 12 distinct highly variable regions, which represent potential molecular markers for *Artocarpus*. These results not only enhance our understanding of the phylogenetic relationships within Chinese *Artocarpus* but also underscore the utility of these specific molecular markers for future studies in this genus.

## Graphical abstract

The initial comparative plastome analysis of 10 Artocarpus species in China lays the groundwork for regional evolutionary research. Investigated of inverted repeat (lR) boundary variations, particularly the incomplete duplication of the ycf1 gene, elucidating mechanisms underlying genomic divergence. Identification of 13 genes under positive selection reveals their roles in environmental adaptation and functional regulation. Constructed highly supported phylogenetic trees using plastome and protein‐coding genes, establishing two clades within the Artocarpus genus. Assessed genetic diversity and identified hypervariable regions (mutation hotspots), providing new avenues for Artocarpus species identification and systematic studies.

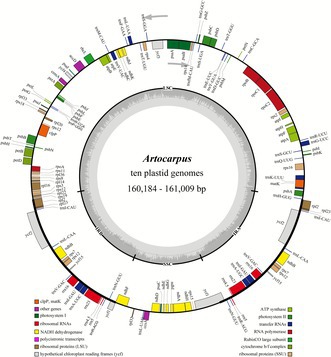

## 1 Introduction

The genus *Artocarpus* J. R. Forst. & G. Forst., which belongs to the Moraceae family, includes about 70 species that primarily thrive in the tropical areas of South and Southeast Asia, along with Oceania (Williams et al. 2017; Zerega et al. 2010). In China, approximately 15 species and two subspecies have been identified. Many species in this genus were known for their significant edible and medicinal qualities. Various *Artocarpus* species have been acknowledged as vital nutritional sources for a range of forest‐dwelling animals (Campbell‐Smith et al. 2011; Sekar and Sukumar 2015). Approximately 12 species, such as jackfruit ( *A. heterophyllus* ), breadfruit ( *A. altilis* ), champedak ( *A. integer* ), dugdug ( *A. mariannensis* ), and breadnut (*A. camansi*), were cultivated as key agricultural enterprises in their respective regions (Witherup et al. 2013; Zerega et al. 2010). Among these, jackfruit was particularly notable, as it was widely grown around the world and was celebrated as a “superfood” due to its outstanding nutritional benefits (Natta et al. 2023). Additionally, certain species of *Artocarpus* were categorized as vulnerable on the IUCN Red List of threatened species. Moreover, *A. gongshanensis*, a plant species with an extremely small population, is a critically protected plant in Yunnan Province due to its narrow distribution and fragmented habitat.

DNA molecular markers, such as *mat*K, *rbc*L, *trn*H‐*psb*A, and ITS, among others, or their combinations, were utilized to assist in species identification. Nonetheless, certain plant taxa with intricate evolutionary backgrounds may not be distinctly distinguished by these markers (Zhang, Zhang, et al. 2025). In *Artocarpus*, early research showed that despite using a combination of DNA fragments (*rbc*L, *mat*K, *trn*L‐*trn*F, *trn*H‐*psb*A, *trn*S‐G, *trn*V‐*ndh*C, ITS, and G3pdh), species complexes with highly similar morphologies and significant taxonomic disputes (e.g., *A. nitidus* and *A. lacucha*) remain ambiguously defined the species (Williams et al. 2017). With the development of next‐generation sequencing (NGS) technologies, it became easier and more cost‐effective to obtain large amounts of genomic data. Plastomes can provide more extensive and higher‐resolution information, and were utilized to resolve phylogenetic relationships, infer genetic connections, and the clarification of taxonomic classification (Li, Luo, et al. 2021; Zhang et al. 2022). Plastids serve as crucial semi‐autonomous organelles found in green plants and specific types of algae. These organelles were primarily responsible for facilitating photosynthesis and various essential metabolic pathways vital for the growth and development of plants (Daniell et al. 2016; Sierra et al. 2023). In higher plants, the structure of the plastome was typically characterized by a quadripartite arrangement, which includes a large single‐copy region (LSC), a small single‐copy region (SSC), and two inverted repeat regions (IRs) (Jansen et al. 2005). The size of plastomes can vary significantly, ranging from 107 to 218 kb, and they typically encompass about 110 to 130 genes that are directly involved in photosynthesis, gene expression, and protein synthesis (Zhang, Zhang, et al. 2020). When compared to nuclear and mitogenome, plastomes evolve at a moderate evolutionary rate and possess a smaller molecular weight. The structural composition of plastomes was relatively conservative, and they were mainly inherited through uniparental means with infrequent recombination occurring (Clegg et al. 1994; Raubeson et al. 2007). These characteristics make plastomes particularly well‐suited for conducting research in plant genetics and evolution. Their utility was widely recognized in various scientific domains, including population genetic diversity, phylogenetic relationships, and biogeographic histories (Clegg et al. 1994; Daniell et al. 2016).

Recent advancements in genomic technology have expedited research on the plastomes of various *Artocarpus* species, such as *A. heterophyllus* (Liu et al. 2018), *A. nanchuanensis* (Li and Song 2019), *A. hypargyreus* (Li et al. 2020), *A. camansi* (Souza et al. 2020), *A. champeden* (Niu and Liu 2021), *A. altilis* (De Souza et al. 2021), *A. gomezianus* (Lin et al. 2021), *A. petelotii* (Chen and Liu 2021), *A. altilis* (Wei et al. 2023), and *A. heterophyllus* var. *seedless* (Ho et al. 2025). In the analysis of phylogenetic relationships based on plastomes, multiple studies confirm that *Artocarpus* and *Morus* are well‐supported sister clades, comprising the core clade in the Moraceae family. These genera represent separate evolutionary lineages with *Ficus* and *Broussonetia*, supporting the monophyly of Moraceae and the divergence relationships among its main internal clades (Liu et al. 2018; Li and Song 2019; Li et al. 2020; Chen and Liu 2021; De Souza et al. 2021; Lin et al. 2021; Niu and Liu 2021; Wei et al. 2023). On the other hand, analysis of plastome phylogenomics indicated that *A. camansi* and *A. altilis* constituted a monophyletic clade with notably close genetic resemblance, providing strong evidence for the theory that *A. camansi* served as the ancestral species for *A. altilis* domestication (Souza et al. 2020).

Plastomes contributed to studies of plant diversification, biogeography, and species interactions (Zhang et al. 2022). Comparative analyses of plastomes not only clarified phylogenetic relationships among species but also identified mutational hotspots in species and provided plastid‐specific markers for subsequent phylogenetic studies and species identification. Additionally, these comparisons assessed genetic variation rates among species at the plastid‐genome level, providing multiple perspectives on species‐level genetic diversity (Li, Huang, Wei, et al. 2025). Unfortunately, there have been no reports about *Artocarpus* species in China. In this study, we presented a comprehensive analysis of the plastomes of 10 *Artocarpus* species from China. Our results refined the understanding of *Artocarpus*' evolutionary history and geographic distribution in China, and provided a high‐value dataset to support future investigations in plant systematics and conservation biology.

## 2 Materials and Methods

### 2.1 Species Collection, Sequencing, Assembly, and Annotation

Ten species were collected in China (Table 1), including *A. heterophyllus* , *A. altilis* , *A. gomezianus*, *A. gongshanensis*, *A. hypargyreus*, *A. lacucha*, *A. nanchuanensis*, *A. nitidus* subsp. *Griffithii*, *A. petelotii*, and *A. tonkinensis* . We obtained plastomes of eight species from the NCBI database, one species from the LCGDB database, and *A. gongshanensis* was newly sequenced. For the newly sequenced *A. gongshanensis*, fresh leaves were collected in the Gaoligong Mountain region of Yunnan, China, and deposited in the Lushui Bureau of the Gaoligong Mountains National Nature Reserve. The genomic DNA was extracted by utilizing a modified CTAB method (Doyle and Doyle 1987). High‐quality DNA that conformed to the criteria for library construction was chosen for sequencing. A paired‐end library with a 500 bp insert size was constructed and sequenced using the Illumina HiSeq 250 platform, resulting in over 5 GB of raw data.

**TABLE 1:** Summary of plastome features of 10 *Artocarpus* species in China.

Genome feature		*A. altilis*	*A. gomezianus*	*A. gongshanensis*	*A. heterophyllus*	*A. hypargyreus*	*A. lacucha*	*A. nanchuanensis*	*A. nitidus* subsp. *griffithii*	*A. petelotii*	*A. tonkinensis*
Accessions		MZ929417	MW837773	PV740811	MK303549	MN720648	ON881696	LAU10104	ON881585	MW250918	MZ379793
Length (bp)	Genome	160,184	160,743	160,662	160,389	160,952	160,677	160,752	160,623	161,009	160,987
	LSC	88,791	89,241	89,232	89,077	89,476	89,492	89,345	89,330	89,552	89,551
	IR	25,734	25,691	25,702	25,708	25,703	25,631	25,693	25,633	25,682	25,682
	SSC	19,925	20,120	20,026	19,896	20,070	19,923	20,021	20,027	20,093	20,072
GC content	Genome	36.00%	35.81%	35.75%	36.05%	35.80%	35.78%	35.83%	38.81%	35.79%	35.79%
	LSC	33.70%	33.44%	33.35%	33.70%	33.41%	33.36%	33.45%	33.41%	33.40%	33.40%
	IR	42.72%	42.75%	42.75%	42.80%	42.74%	42.78%	42.76%	42.77%	42.76%	42.76%
	SSC	28.84%	28.58%	28.47%	29.17%	28.63%	28.63%	28.65%	28.68%	28.62%	28.63%
	AT‐skew	−0.015	−0.015	−0.014	−0.015	−0.014	−0.013	−0.015	−0.014	−0.014	−0.014
	GC‐skew	−0.016	−0.017	−0.016	−0.017	−0.017	−0.016	−0.017	−0.017	−0.017	−0.017
Gene number	Genome	132	132	132	132	132	132	132	132	132	132
	CDS	87	87	87	87	87	87	87	87	87	87
	tRNA	37	37	37	37	37	37	37	37	37	37
	rRNA	8	8	8	8	8	8	8	8	8	8
Sources		NCBI	NCBI	Newly sequenced	NCBI	NCBI	NCBI	LCGDB	NCBI	NCBI	NCBI

Abbreviations: LCGDB, Lauraceae Chloroplast Genome Database; NCBI, National Center for Biotechnology Information.

Raw reads involved eliminating low‐quality reads and adapters with Fastp (Chen et al. 2018) to generate high‐quality clean reads for further analysis. Subsequently, the circular plastome was assembled with GetOrganelle (Jin et al. 2020) and evaluated using Bandage (Wick et al. 2015) for *A. gongshanensis*. In order to ensure the structural and orientational consistency of the plastome of *A. gongshanensis* with other *Artocarpus* species, we aligned the assembled plastome with other *Artocarpus* species and conducted collinearity checks employing Mauve (Darling et al. 2004) using default settings. Annotation of the plastome was conducted through CPGAVAS2 online (Shi et al. 2019) with reference to *A. heterophyllus* (MK303549), followed by manual adjustments in Geneious (Kearse et al. 2012) to precisely delineate the start/stop codons, as well as the intron/exon boundaries of protein‐coding genes. Finally, a circular structural diagram of the complete genome was generated by Organelle Genome DRAW (OGDRAW) (Lohse et al. 2013). The fully annotated plastome sequence was submitted to the NCBI database, and its accession number is PV740811.

### 2.2 Repeat Sequence Analysis

The MicroSAtellite Identification Tool (MISA, Beier et al. 2017) determined the minimum repeat thresholds for various nucleotide motifs in the 10 Artocarpus plastomes, with the thresholds set at 10 for mononucleotides, five for dinucleotides, four for trinucleotides, three for tetranucleotides, pentanucleotides, and hexanucleotides. Simple sequence repeats (SSRs) ranging from 1 to 10 units were identified within these plastomes. Furthermore, REPuter software (Kurtz et al. 2001) was employed to detect complementary repeats, forward repeats, palindromic repeats, and reverse repeats in the non‐simple sequence repeats (non‐SSRs) of 10 plastomes, using parameter values of a hamming distance of three, a maximum of 5000 computed repeats, and a minimal repeat size of 30.

### 2.3 Codon Preference, IR Boundary Analyses, and Comparative Genome Analysis

Protein‐coding genes were extracted from the plastomes of 10 Artocarpus species using PhyloSuite software (Zhang, Gao, et al. 2020), and genes that were shorter than 300 bp and redundant were excluded. CodonW (Romero 2000) was utilized to calculate the relative synonymous codon usage (RSCU) for the remaining coding genes. Inverted repeat (IR) boundary contraction and expansion were analyzed by performing CPJSdraw (Li et al. 2023). Additionally, to uncover plastomes' differentiation and mutation hotspots, the mVISTA online tool (Frazer et al. 2004) was employed under the Shuffle‐LAGAN model.

### 2.4 Evaluation of Selective Pressure Regimes

Substitution rates for synonymous (Ks) and non‐synonymous (Ka) were calculated for 72 protein‐coding genes found in 10 *Artocarpus* species in China. The genes were extracted through PhyloSuite (Zhang, Gao, et al. 2020) and subsequently aligned using MAFFT (Katoh and Standley 2013). The nucleotide substitution rates of Ka and Ks, along with the Ka/Ks ratio, were determined employing KaKs_Calculator (Zhang 2022) by the YN model. The interpretation of the Ka/Ks ratio followed standard thresholds: Ka/Ks > 1 indicates positive selection, Ka/Ks = 1 indicates neutral selection, 0.5 < Ka/Ks < 1 indicates relaxed selection, and Ka/Ks < 0.5 indicates purifying selection (Kimura 1989).

### 2.5 Phylogenetic Reconstruction

To elucidate the phylogenetic connections within the *Artocarpus* genus, one new plastid genome was sequenced, and 41 plastid genomes were obtained from the NCBI and LCGDB databases (https://lcgdb.wordpress.com/), including 23 *Artocarpus* individuals, six individuals from *Maclura*, 10 individuals from *Ficus* L., two individuals from *Antiaris*, and *Morus wittiorum* as outgroups. Two datasets, which included plastid genomes and protein‐coding genes, were aligned using MAFFT (Katoh and Standley 2013) and subsequently refined manually with BioEdit (Hall 1999). The GTR + I + G substitution model was identified as the most suitable model for both datasets through analyses conducted with jModelTest (Darriba et al. 2012). Subsequently, phylogenetic trees were constructed employing both maximum‐likelihood (ML) and Bayesian inference (BI) methods. The ML analysis was executed by IQ‐TREE (Nguyen et al. 2015), involving 1000 bootstrap resampling replicates, which were evaluated using the Bayesian information criterion (BIC) to assess branch support. For the BI analysis carried out with MrBayes (Ronquist and Huelsenbeck 2003), the Markov Chain Monte Carlo (MCMC) algorithm was run for 1,000,000 generations, sampling every 1000 generations until the average standard deviation of split frequencies fell below 0.01. The initial 25% of trees produced were excluded during the burn‐in phase, with the remaining individuals employed to build a consensus tree under majority rule. All phylogenetic trees were subsequently visualized and optimized using FigTree (https://tree.bio.ed.ac.uk/software/figtree/).

### 2.6 Sliding Window Analysis

Initially, one matrix for 10 *Artocarpus* species in China was derived from the adjusted matrix. Subsequently, the sequence matrix corresponding to Clade A and Clade B was extracted according to the results of the phylogenetic analyses. The nucleotide diversity values (*π*) for each matrix were calculated using DnaSP (Rozas et al. 2017), employing a window length of 600 bp and a step size of 200 bp.

## 3 Results

### 3.1 Characteristics of the Plastomes of 10 Artocarpus Species From China

**FIGURE 1:**
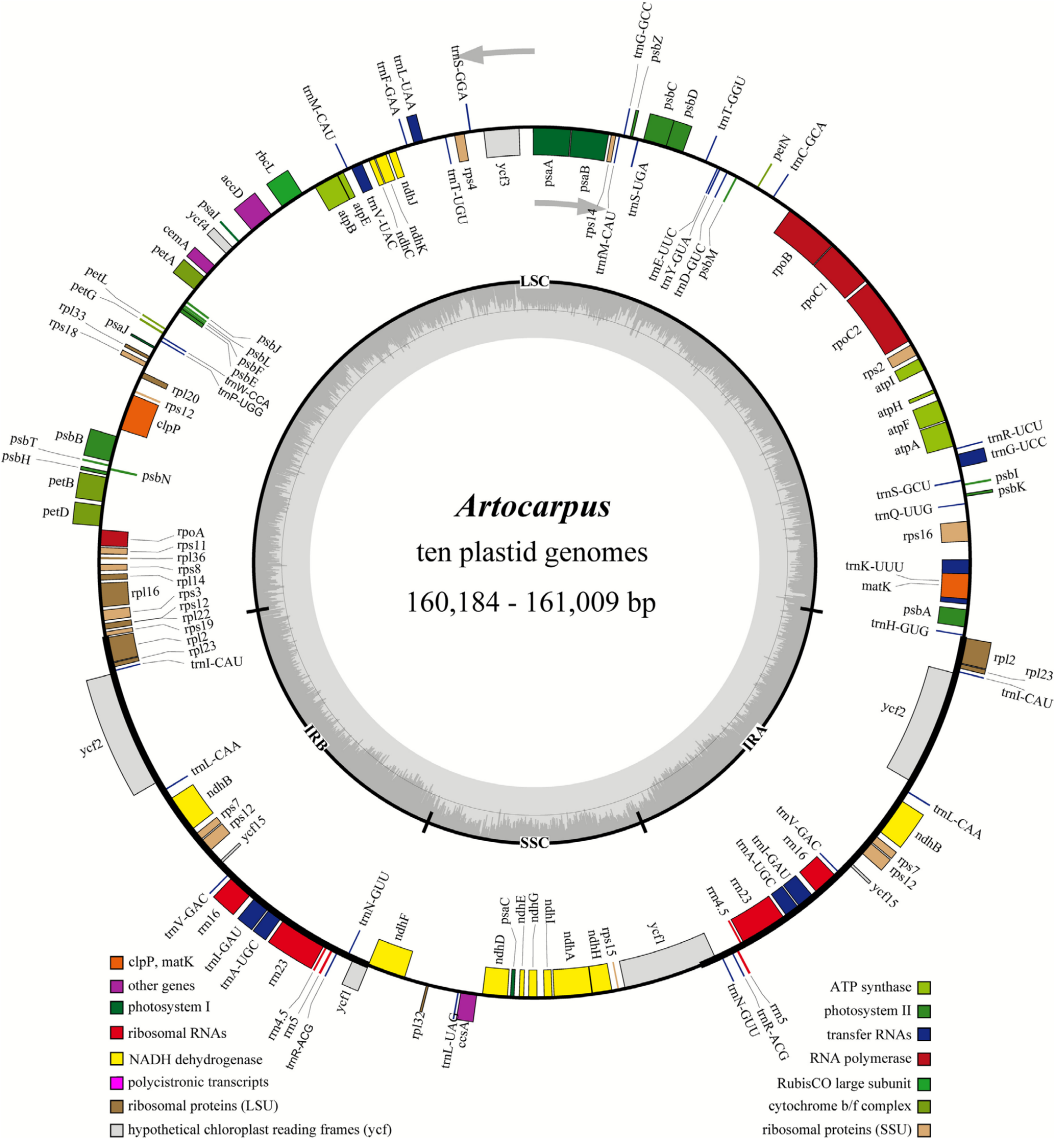
Circular map of plastomes from ten Chinese *Artocarpus* species. Genes inside and outside the circle are transcribed clockwise and counterclockwise, respectively. Genes from different functional groups are color‐coded. Inner circle: Dark gray represents GC content, and light gray represents AT content.

The circular, double‐stranded structure of plastid genomes of 10 *Artocarpus* species in China exhibited sizes varying from 160,184 bp for *A. altilis* to 161,009 bp for *A. petelotii*, with guanine‐cytosine (GC) content ranging from 35.75% to 38.81% (Figure 1, Table 1). The genomes of these plastid genomes displayed a characteristic quadripartite structure, which includes one LSC region, one SSC region, and two IRs. The lengths of the LSC region ranged from 88,791 bp to 89,552 bp, with a GC content that varied between 33.35% and 33.70%. The SSC region measured between 19,896 bp and 20,120 bp, displaying a GC content of 28.63% to 29.17%. The IR regions ranged from 25,631 bp to 25,734 bp, showing a GC content from 42.72% to 42.80%. Although there were minor differences in the sizes of the plastomes, analyses revealed several conserved genetic characteristics across the 10 Chinese *Artocarpus* species. In total, 132 genes were identified, including 87 protein‐coding genes, 37 tRNA genes, and 8 rRNA genes.

### 3.2 SSR and Repeat Analyses

**FIGURE 2:**
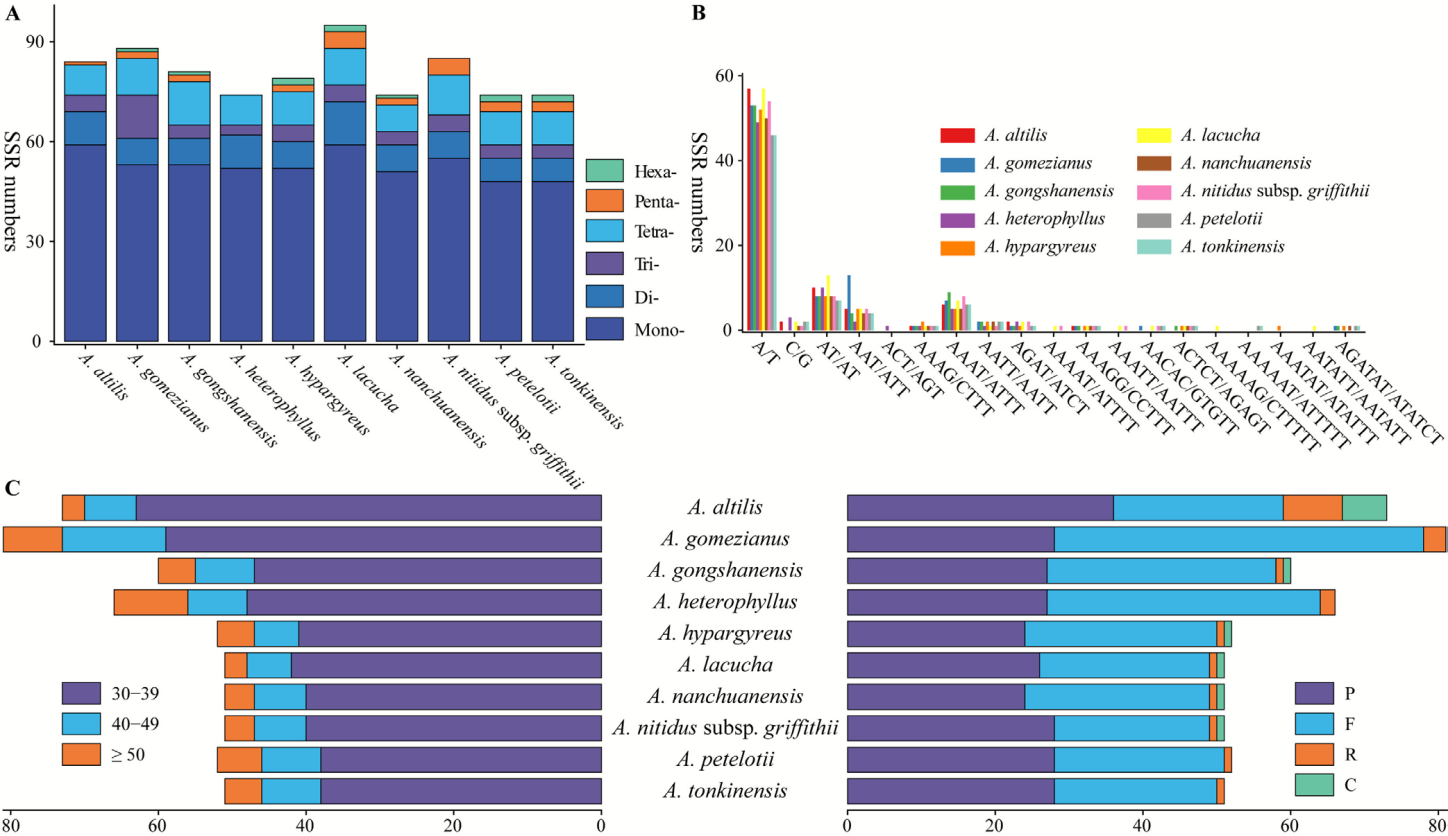
Types and counts of repeats. (A) Frequency of different SSR types. (B) Frequency of SSR motifs across distinct repeat classes. (C) Types and numbers of dispersed repeats. Left panel: Distribution of repeats by length; right panel: Distribution of repeat types. C, complement repeats; F, forward repeats; P, palindromic repeats; R, reverse repeats.

The examination of the 10 plastomes revealed between 73 and 95 simple sequence repeats (SSRs), which exhibited lengths ranging from mononucleotide to hexanucleotide repeats, but not all six types of repeats were detected (Figure 2A). Mononucleotide repeats were the most prevalent, representing 60.23% to 70.27% of all SSRs (Figure 2A, Table S1). In all mononucleotide repeats, the A/T repeat type was predominant, but only zero to two C/G repeat types were identified. Notably, *A. heterophyllus* showed no pentanucleotide repeats, and *A. altilis* , *A. heterophyllus* , and *A. nitidus* subsp. g*riffithii* lacked hexanucleotide repeats (Figure 2B, Table S1).

Furthermore, in the plastid genomes of the 10 species of *Artocarpus*, four distinct types of dispersed repeats were detected, including forward, reverse, complement, and palindromic repeats, with totals ranging from 51 (in *A. lacucha*, *A. nanchuanensis*, *A. nitidus* subsp. *griffithii*, and *A. tonkinensis* ) to 81 (in *A. gomezianus*) (Table S2). The complementary repeats of forward and palindromic were predominant, while the occurrence of reverse repeats varied from one to eight among these repeats (Figure 2C), and complement repeats were not observed in *A. gomezianus*, *A. heterophyllus* , *A. petelotii*, and *A. tonkinensis* . The lengths of the repeats were primarily found within the range of 30–39 bp, showing significant variation. Additionally, the 40–49 bp range contained between 6 and 14 repeats, while there were between 3 and 10 repeats that exceeded 50 bp (Figure 2C).

### 3.3 Codon Usage Bias Analysis

**FIGURE 3:**
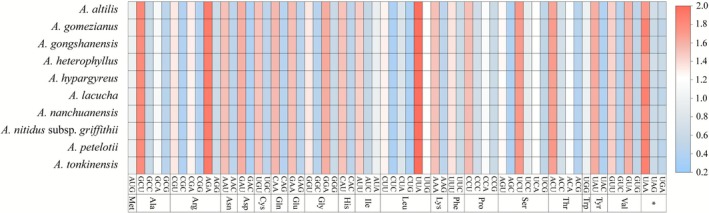
A heatmap illustrating the RSCU values for China 10 species of *Artocarpus*.

The RSCU patterns in protein‐coding genes across 10 species of *Artocarpus* species showed similar frequencies with minor variations. These genes contain 20,697 and 21,804 codons, which can be categorized into 64 unique codons that encode 21 amino acids (Figure 3, Table S3). An RSCU value greater than 1.00 indicates a preference for that corresponding codon, while a value less than 1.00 indicates a lower frequency of usage. Out of the 64 codons investigated, 30 showed RSCU values greater than 1, with 13 ending in A, 16 in U, and one in G, suggesting a preference for A or U endings. The codons AGA, GCU, and UUA have the highest RSCU values, all surpassing 1.8, corresponding to the amino acids Arginine, Alanine, and Leucine, respectively. In contrast, the RSCU values for AUG and UGG, which code for Methionine and Tryptophan, were 1.0, reflecting no preference in usage. Notably, AUG was frequently used as the start codon during translation, signaling the beginning of protein synthesis. The codons CUC, CUG, and AGC, encoding Leucine and Serine, exhibit the lowest RSCU values and were consistently observed across the 10 plastomes examined.

### 3.4 IR Region Contraction and Expansion

**FIGURE 4:**
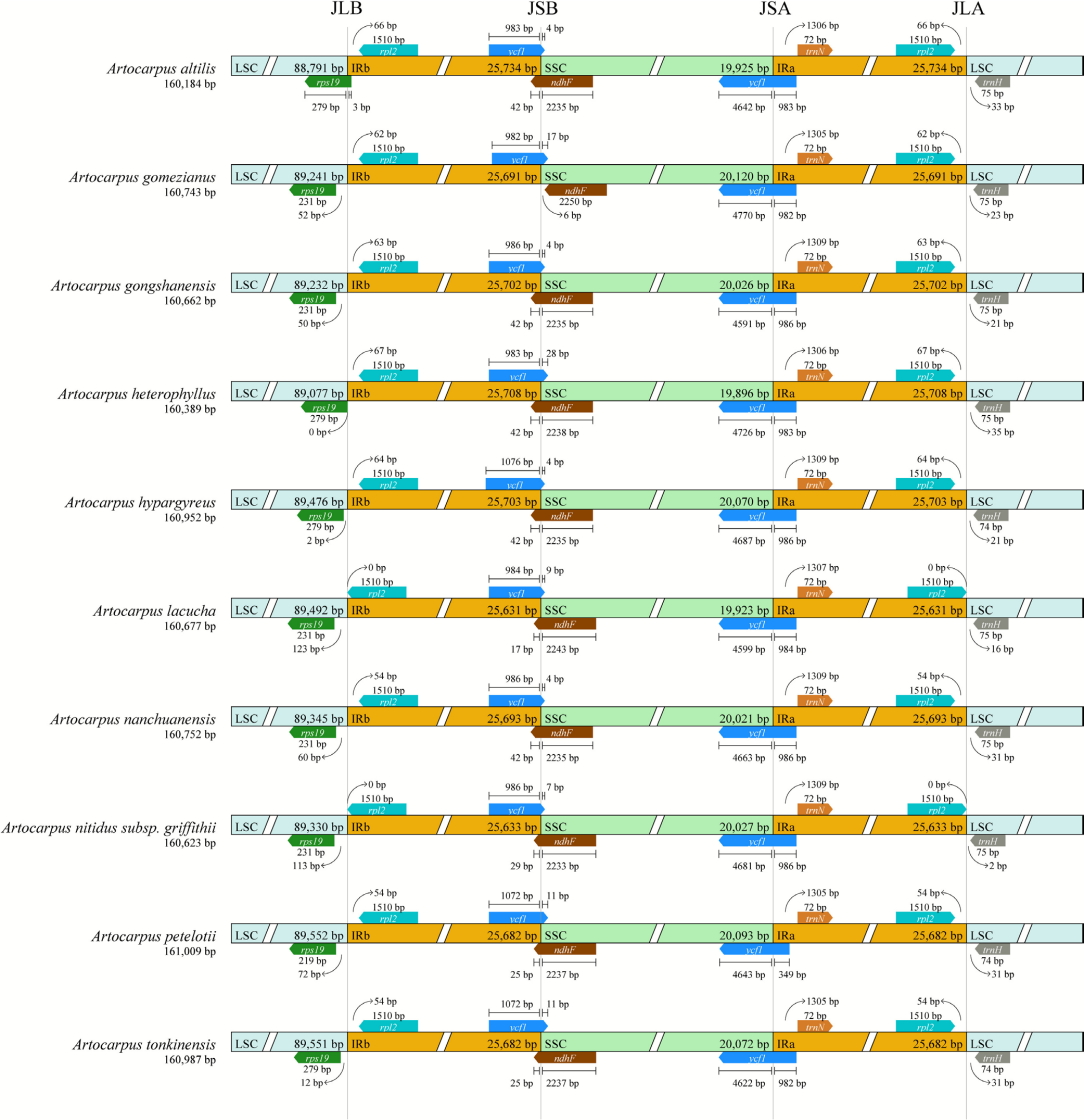
A comparative analysis of the junction positions of LSC, IR, and SSC regions across 10 plastomes of Artocarpus species from China.

The study investigated the boundaries of inverted repeat (IR) in 10 *Artocarpus* species from China, focusing on potential expansions or contractions within these regions. The genes *ycf1* and *ndhF* were precisely positioned at these boundaries, while *rps19*, *rpl2*, *ndhF*, and *trnN* were adjacent to the boundary regions (Figure 4). Genes like *rpl2* and *trnN, not* located at the boundaries, have their lengths conserved, but their distances from the boundaries differ. On the other hand, genes located at the boundaries showed different degrees of expansion or contraction. This variability was noted not only among various genomes but also across different boundaries within an individual genome. Particularly, the *ycf*1 genes displayed significant incomplete duplication, with the primary segment of *ycf*1 found at the JSA boundary located in the SSC region, whereas the *ycf*1 at the JSB boundary had primarily lost its segment in the SSC region.

### 3.5 Analysis of Selective Evolutionary Pressure

**FIGURE 5:**
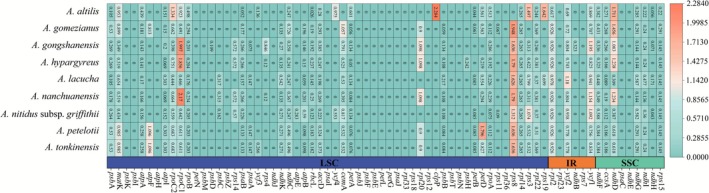
Selective pressure distribution across 10 *Artocarpus* plastomes from China. It displays Ka/Ks ratios for 72 protein‐coding genes conserved across *A. heterophyllus* and nine congeneric species.

A comparative analysis of 72 protein‐coding genes shared among 10 *Artocarpus* species in China revealed significant variations in evolutionary constraints (Figure 5). Utilizing the plastome of *A. heterophyllus* as a reference, this research assessed the ratio of Ka to Ks substitution rates for a total of 72 conserved protein‐coding genes. The analysis produced an average Ka/Ks ratio of 0.23, indicating that purifying selection predominantly influenced plastome evolution. This finding implied robust evolutionary constraints that contribute to upholding the functional stability of most plastome genes.

In addition, the analysis revealed specific genes exhibiting divergent evolutionary patterns across the *Artocarpus* species examined. Notably, the *atp*F gene in *A. petelotii* and *A. tonkinensis* , the *rpo*C2 gene in *A. altilis* , the *rpo*C1 gene in *A. gongshanensis*, *A. hypargyreus*, and *A. nanchuanensis*, the *cem*A gene in *A. gomezianus*, the *rpl*20 gene in *A. gongshanensis*, *A. hypargyreus*, and *A. nanchuanensis*, the *clp*P gene in *A. altilis* , the *pet*D gene in *A. petelotii*, the *rps*8 gene in *A. gomezianus*, *A. gongshanensis*, *A. hypargyreus*, *A. lacucha*, *A. nanchuanensis*, *A. nitidus* subsp. *griffithii*, *A. petelotii*, and *A. tonkinensis* , the *rps*3 gene in *A. altilis* and *A. nitidus* subsp. *griffithii*, the *rps*19 gene in *A. altilis* , the *ycf*2 gene in *A. lacucha*, and the *ycf*1 gene in *A. gongshanensis*, *A. nanchuanensis*, and *A. nitidus* subsp. *griffithii*, as well as the *ndh*D gene in *A. altilis* , *A. gomezianus*, *A. gongshanensis*, *A. hypargyreus*, and *A. nanchuanensis*, exhibiting Ka/Ks ratios above 1.0, suggesting potential positive selection. Conversely, the other genes consistently exhibited Ka/Ks ratios below 1.0, supporting purifying selection as the primary evolutionary force in *Artocarpus* plastomes. This pattern of selective pressure indicates that most plastome genes retain critical functions with high interspecific conservation, while certain genes have experienced adaptive evolution.

### 3.6 Phylogenetic Analyses

Comprehensive phylogenetic analyses were carried out to determine the phylogenetic placement of *Artocarpus* within the Artocarpoideae subfamily. Plastomes and protein‐coding genes from this subfamily were utilized, and *M. wittiorum* was assigned as the outgroup. The plastomes and protein‐coding genes had alignment lengths of 176,129 bp and 92,067 bp, containing 22,814 and 5648 variable sites, and 13,349 and 4543 parsimony informative sites, respectively.

**FIGURE 6:**
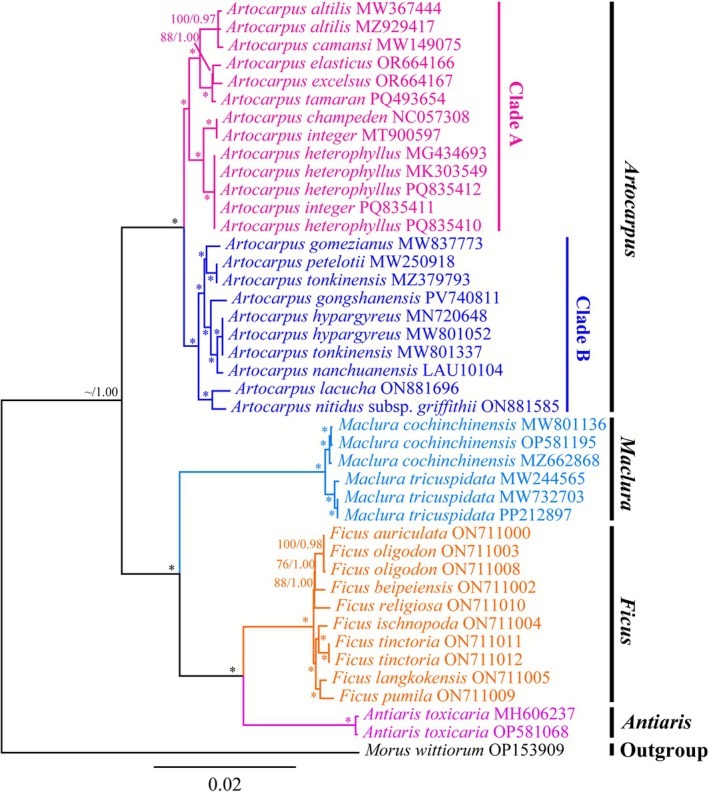
Phylogenetic tree of 42 taxa from the subfamily Artocarpoideae, constructed using plastomes via Bayesian inference (BI) and maximum likelihood (ML). Values at each node indicate bootstrap support. Branch‐associated values represent BI posterior probabilities (PP) and ML bootstrap values (BS); asterisks (*) indicate bootstrap values/posterior probabilities of 100/1.00.

ML and BI analyses were consistent, offering robust evidence for the phylogenetic relationships (Figures 6 and S1). In addition, the phylogenetic trees constructed from both plastid genomes and protein‐coding genes exhibited consistent topological structures (Figures 6 and S1), dividing *Artocarpus* species into two clades with high support values at the key nodes (MLBP/PP = 100/1.00, Figures 6 and S1). The subfamily Artocarpoideae was further delineated into four groups, including *Antiaris* Lesch., *Ficus* L., *Maclura* Nutt., and *Artocarpus*. In the *Artocarpus* group, Clade A includes *A. heterophyllus* , *A. integer* , *A. champeden* , *A. tamarin* Becc., *A. excelsus* F.M.Jarrett, *A. camansi* Blanco, and *A. altilis* (Figures 6 and S1). Clade B includes *A. nitidus* subsp. *griffithii*, *A. lacucha*, *A. nanchuanensis*, *A. tonkinensis* , *A. hypargyreus*, *A. gongshanensis*, *A. tonkinensis* , *A. petelotii*, and *A. gomezianus* (Figures 6 and S1).

### 3.7 Identification of Mutational Hotspots

The analysis of genome differentiation and mutation hotspots, evaluated using mVISTA software, demonstrated a substantial level of similarity and significant conservation across the 10 *Artocarpus* species from China. Divergence was notably more prominent in the single‐copy regions than in the IRs. Additionally, non‐coding regions exhibited greater divergence than the coding regions (Figure S2).

**FIGURE 7:**
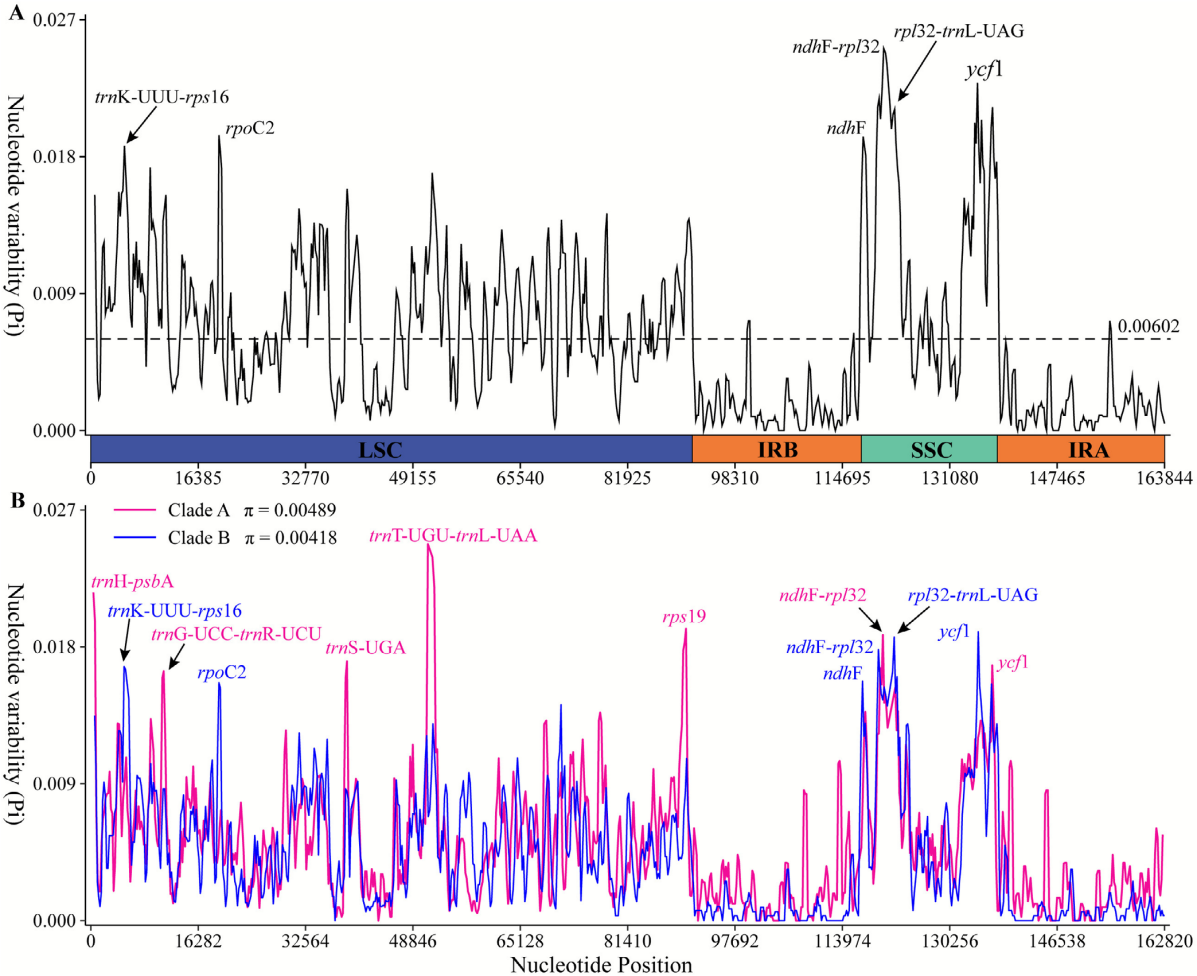
Nucleotide variability (*π*) across 10 *Artocarpus* plastomes from China. (A) Overall *π* values; (B) *π* values for Clade A and Clade B. *x*‐axis: Window midpoint position; *y*‐axis: Window‐specific nucleotide diversity.

Genetic diversity (*π*) was assessed in 10 *Artocarpus* species from China, as well as on Clade A and Clade B. The *π* values for these species ranged between 0 and 0.02511, averaging 0.00602 (Figure 7A). Six regions with highly variable *π* were pinpointed (*π* > 0.018), namely *trn*K‐UUU‐*rps*16, *rpo*C2, *ndh*F, *ndh*F*‐rpl*32, *rpl*32‐*trn*L‐UAG, and *ycf*1. Clade A (Figure 6), comprising 13 *Artocarpus* sequences, showed *π* values ranging from 0 to 0.02474, with an average of 0.00489. Seven highly variable regions were identified, including the regions of *trn*H‐*psb*A, *trn*G‐UCC‐*trn*R‐UCU, *trn*S‐UGA, *trn*T‐UGU‐*trn*L‐UAA, *rps*19, *nad*F‐*rpl*32, and *ycf*1 (Figure 7B). Clade B (Figure 6), with 10 sequences, displayed *π* values from 0 to 0.019, averaging 0.00418. Six regions with a high degree of variability were detected, encompassing the regions of *trn*K‐UUU‐*rps*16, *rpo*C2, *nad*F, *nad*F‐*rpl*32, *rpl*32‐*trn*L‐UAG, and *ycf*1 (Figure 7B). The patterns of nucleotide changes in Clade A and Clade B exhibited striking similarity, with considerable overlap in the most significant regions of variation.

The LSC region comprised the seven hypervariable regions, including *trn*H‐*psb*A, *trn*K‐UUU‐*rps*16, *trn*G‐UCC‐*trn*R‐UCU, *rpo*C2, *trn*S‐UGA, *trn*T‐UGU‐*trn*L‐UAA, and *rps*19, while the other regions were found in the SSC region. An anomaly was observed within a segment of the *ycf*1 gene located in the IR region, where no additional high‐variability regions were found. Notably, these highly variable regions were mainly located in intergenic spacers, only five regions (*rpo*C2, *rps*19, *nad*F, *rpl*32, and *ycf*1) being intragenic.

## 4 Discussion

### 4.1 Plastome Structure in Artocarpus

Gene transfer and loss were common in plant plastomes, contributing significantly to plant evolution and adaptation (Li et al. 2017; Martin et al. 2002). In parasitic and hemiparasitic plants, like *Cuscuta* (Braukmann et al. 2013; Pan et al. 2023), *Cassytha* (Song et al. 2017), and *Pedicularis*, gene pseudogenization and loss in plastomes were prominent (Li, Yang, et al. 2021). In this study, analyzing the plastomes of 10 *Artocarpus* species in China revealed significant similarities in structure, size, gene content, and gene order, indicating a high level of conservation (Ho et al. 2025). Among the 10 sequenced plastomes in China, *A*. *petelotii* had the largest genome size at 161,009 bp, while *A. altilis* had the smallest at 160,184 bp, a difference of 825 bp. These genomic differences were attributed to changes across multiple genomic regions, including insertions, deletions, and alterations in inverted repeats (IRs) (Zheng et al. 2017).

SSRs were essential for genomic recombination and rearrangement, and were scattered across the genome (Vieira et al. 2016). Repetitive DNA might serve as the core driving force behind genome evolution, speciation, and ecological adaptation (Kim et al. 2025). SSRs, exhibiting their high polymorphism and significant intraspecific variation, were useful in investigating genetic diversity, population structure, and biogeography in *Artocarpus* species, both inter‐ and intra‐species. In this study of 10 *Artocarpus* species in China, 73 to 95 SSRs were identified within the plastomes, demonstrating variability in their numbers among the different species (Figure 2A). Additionally, this study discovered 51 to 81 larger repeats (Figure 2C), palindromic and forward repeats may contribute to maintaining genomic stability and were associated with genomic rearrangements that promote species diversification (Zhang, Yu, et al. 2025). These SSRs and repeats serve as valuable tools for germplasm authentication and monitoring genetic diversity in *Artocarpus*, thereby supporting conservation efforts for threatened and endangered species in this genus.

The structure of the plastome, specifically the expansion and contraction in the IRs, can indicate the diversity of these genomes and impact the phylogenetic relationships among species (Wang et al. 2008, 2022). Previous research has demonstrated that size variations in plastomes within a genus were associated with the contraction and expansion of the IRs (Ravi et al. 2008). However, an analysis of the plastomes of 10 *Artocarpus* species from China revealed that the gene distribution was consistent across all boundary regions of the IR, with no rearrangement events, which potentially leads to only minor differences in plastome size within this genus. Additionally, it has been confirmed that the *ycf*1 genes were located at the boundaries between the IR region and the large single‐copy (LSC)/small single‐copy (SSC) regions, where incomplete replication occurs (Song et al. 2015).

This study supports this observation, as the length variation of the *ycf*1 gene at the junction boundaries of JSB and JSA shows different levels of expansion and contraction across the plastid genomes of the 10 *Artocarpus* species in China (Figure 4). Furthermore, the *ndh*F gene also demonstrates similar expansion and contraction; comparable gene expansion and contraction have also been observed in ferns (Fan et al. 2021).

### 4.2 Adaptive Evolution in Artocarpus

The Ka/Ks ratio was a widely used metric for assessing natural selection pressures acting on protein‐coding genes (Gonzales et al. 2002; Pond and Muse 2005). In this study, our analyses indicated that most plastome genes undergo purifying selection, with an average Ka/Ks ratio of 0.23 (Figure 5). These findings align with earlier studies demonstrating widespread purifying selection on most plastome genes in different lineages like *Ficus* (Zhang et al. 2022), the Simaroubaceae family (Liu et al. 2025), and Beilschmiediineae trees (Zhu et al. 2024), reflecting their remarkably conserved evolutionary history.

In this study, we identify positive selection in the plastome genes (Figure 5), which were in *atp*F, *rpo*C2, *rpo*C1, *cem*A, *rpl*20, *clp*P, *pet*D, *rps*8, *rps*3, *rps*19, *ycf*2, *ycf*1, and *ndh*D, respectively, indicating their potential involvement in facilitating environmental adaptation in *Artocarpus*. Functionally, these positively selected genes were primarily linked to biological processes like transcription, translation, photosynthesis, and protein metabolism. Notably, the *rpo*C1 and *rpo*C2 genes, which were crucial for transcriptional regulation of gene expression, also underwent positive selection in the Orchidaceae (Mauad et al. 2021) and Salicaceae (Zhou et al. 2021; Zong et al. 2019) families. The genes *rpl*20, *rps*8, *rps*3, and *rps*19 were essential for plastome ribosome assembly and protein synthesis. In addition, the *rps*8 gene was found and also has shown positive selection in eight *Artocarpus* species distributed in China (Figure 5). The *rps*8 gene, the primary function that encodes a small subunit protein of the plastome ribosome, facilitates the synthesis of chloroplast‐encoded proteins and indirectly supports critical physiological processes such as photosynthesis. Positive selection has been noted for the *rps*8 gene in species of *Cotinus* (Liu et al. 2023). The *atp*F, *pet*D, and *ndh*D genes were primarily involved in the light reaction and energy metabolism. *A. heterophyllus* , which primarily inhabits tropical and subtropical regions with significantly fluctuating light intensities, relies on these genes to dynamically regulate photosynthesis and energy metabolism. The *atp*F gene, encoding *atp*F protease, was also shown to be positively selected in angiosperm lineages, such as *Quercus aquifolioides* (Yin et al. 2018). In this study, positive selection was detected in the *ndh*D gene in five *Artocarpus* species ( *A. altilis* , *A. gomezianus*, *A. gongshanensis*, *A. hypargyreus*, and *A. nanchuanensis*). The *cem*A is crucial for carbon metabolism and membrane transport processes, whereas the *clp*P gene is essential for protein degradation and homeostasis maintenance. The *ycf*1 and *ycf*2 genes were likely to contribute to plastome development or transport. Our findings indicate that positively selected plastome functional genes may drive the adaptive radiation and ecological specialization of *Artocarpus* species in terrestrial ecosystems.

### 4.3 Phylogenetic Relationships of the Genus Artocarpus

In previous research, phylogenetic studies of the genus *Artocarpus* typically employed various nuclear and plastid markers (Gardner et al. 2021; Gardner and Zerega 2021; Ho et al. 2025; Williams et al. 2017; Zerega et al. 2010). Nevertheless, employing different markers often led to the formation of distinct phylogenetic trees. Here, a phylogenetic tree was reconstructed based on the plastid genomes and protein‐coding genes, which demonstrated a stable topological structure consisting of two distinct clades. This finding provides new insights into the systematic relationships within *Artocarpus* (Figures 6 and S1). Notably, the phylogenetic tree constructed from the plastomes revealed phylogenetic relationships that aligned closely with Ho's findings, except *A. heterophyllus* (MK303549.1) (Ho et al. 2025). However, using Target Capture Sequencing (HybSeq), researchers obtained sufficiently long loci, and the phylogenetic trees constructed with single‐copy genes differ from those in our study, and *Artocarpus* was divided into four subgeneric clades, with each subgenus being monophyletic (Gardner et al. 2021; Gardner and Zerega 2021). In other words, the phylogenetic tree based on the plastomes may not completely reflect the phylogenetic relationships due to plastomes being maternally inherited and dominating the evolutionary history, excluding the paternal contribution (Li, Lu, Qin, et al. 2025). Additionally, the evolutionary rates of plastomes were heterogeneous, which made it difficult to accurately resolve deep‐level or recent phylogenetic relationships. This was attributable to variations in plastome evolutionary speeds among different taxa, with some plastomes being excessively conserved or evolutionarily saturated (Zhang, Sun, et al. 2020). Moreover, the plastome exhibits horizontal gene transfer, recombination, incomplete lineage sorting, hybridization, and plastid capture, which complicated its evolutionary history, and caution should be exercised when using the plastome data to elucidate the phylogenies (Stull et al. 2020; Cauz‐Santos 2025; Li, Lu, Antonelli, et al. 2025; Wu et al. 2025; Zhang, Zhang, et al. 2025). Therefore, it is crucial to exploit more nuclear data, such as nrDNA (Zhang, Zhang, et al. 2025), RAD data (Ding et al. 2019), single‐copy fragments obtained through Hyb‐seq (Gardner et al. 2021; Gardner and Zerega 2021), deep genome skimming (Lin et al. 2025), single‐copy orthologous genes obtained from transcriptome sequencing (Wei et al. 2024), and whole genome resequencing (Yi et al. 2025), for uncovering the complicated phylogenetic relationships. In addition, our research focused exclusively on the plastomes of 10 *Artocarpus* species distributed in China and without encompassing a broader global representation of the genus or the other five species native to China. This restriction affected the geographic representativeness of our samples and limited overall species coverage. As a result, it became difficult to generalize our conclusions to the global diversity of *Artocarpus* worldwide. Consequently, these limitations may limit the comprehensiveness of our evolutionary inferences about the genus. Future research aimed at expanding global sampling and integrating nuclear and mitochondrial data to resolve phylogenetic conflicts.

**FIGURE 8:**
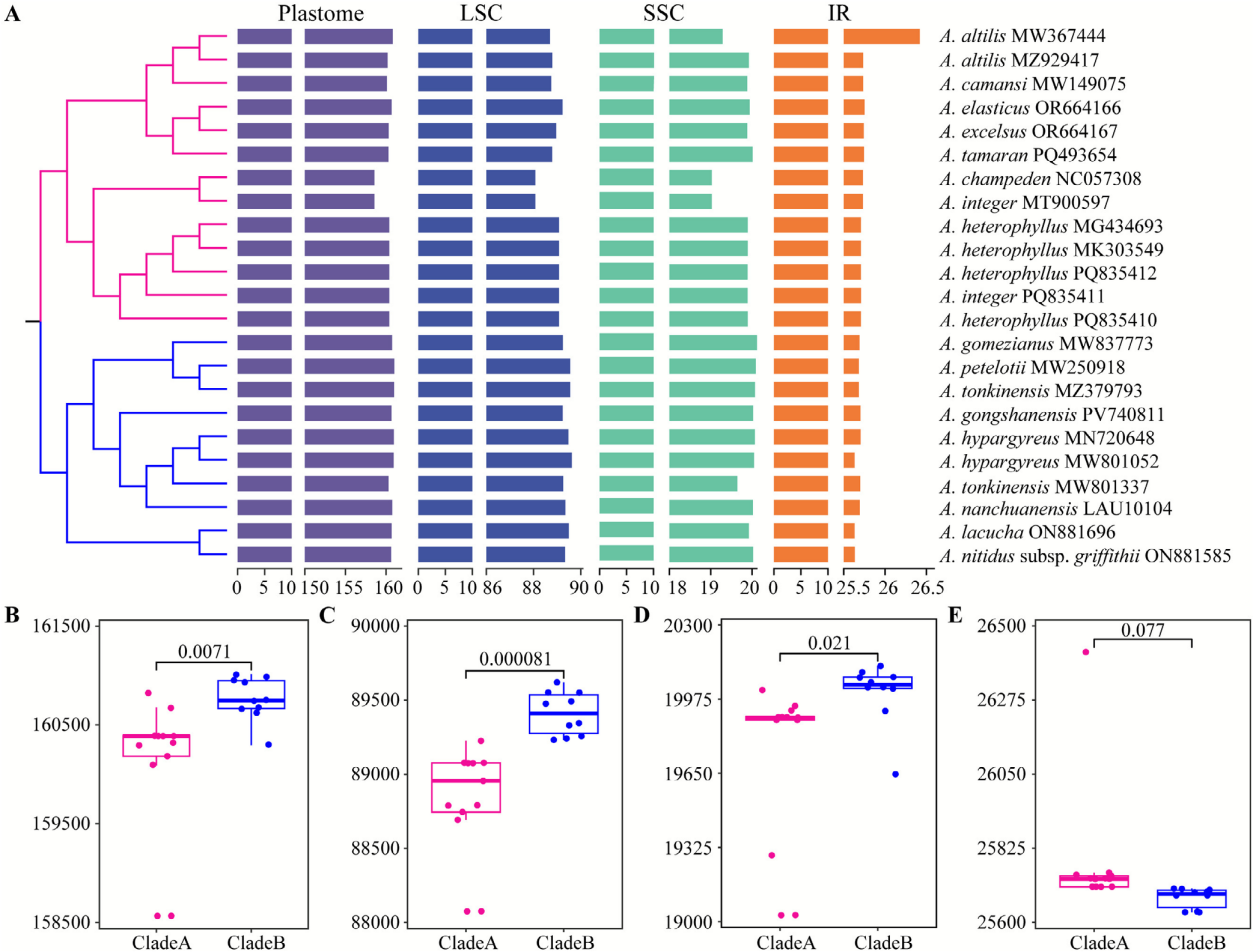
Plastome length comparison between Clade A and Clade B. (A) Lengths of distinct genomic regions mapped onto the phylogenetic tree (red branches = Clade A; blue branches = Clade B). (B) Total plastome length comparison. (C) LSC region length comparison. (D) IR region length comparison. (E) SSC region length comparison.

Notably, all species in Clade B were native to China, while Clade A includes the cultivated species *A. altilis* and *A. heterophyllus* found in China. Interestingly, the branches of *A. integer* and *A. heterophyllus* were interlaced within one another, suggesting the presence of intraspecific genetic variations or differences among distinct geographical populations within these species. Comparing plastome sizes of species within Clade A and Clade B on their phylogenetic trees reveals significant differences in genome sizes. Phylogenetic trees found that the plastomes of Clade B species were larger than those of Clade A (Figure 8A,B). Research indicates a positive correlation between the synonymous substitution rate and genome size in angiosperm plastomes. Larger genome sizes were typically associated with more non‐coding regions, where mutations can accumulate at an accelerated rate, potentially promoting species diversification (Bromham et al. 2015). This pattern was similarly observed in coniferous species (Wu and Chaw 2016), indicating that species in Clade B may have experienced more rapid evolutionary divergence compared to those in Clade A. Further comparative analyses of the plastome lengths and their lengths of distinct regions, including the LSC, SSC, and IRs, revealed significant disparities between Clades A and Clades B (Figure 8C–E). Similarly, in Asiatic core *Beilschmiedia* populations, variations in IR and SSC regions were likely the primary contributors to genome length differences (Zhu et al. 2025). In *Artocarpus* species, variations across these three regions were likely the main drivers of overall genome length differences.

### 4.4 Analysis of Divergence Hotspots

Genetic mutations do not always occur randomly, but certain ones were known to cluster in specific areas (Shaw et al. 2007; Worberg et al. 2007), leading to highly variable genome regions (Song et al. 2015). These highly variable regions were frequently utilized as barcoding markers for taxon identification and phylogenetic analyses (Hollingsworth et al. 2009). In this study, species of *Artocarpus* were classified into two groups: Clade A and Clade B. Consequently, we examined variations in the plastome across these clades to discover potential molecular markers that differentiate them, ultimately identifying five promising regions: *trn*H‐*psb*A, *trn*G‐UCC‐*trn*R‐UCU, *trn*S‐UGA, *trn*T‐UGU‐*trn*L‐UAA, and *rps*19. By integrating the variation regions observed in Clade A and Clade B, we identified 11 highly variable regions across the two clades, including *trn*H‐*psb*A, *trn*K‐UUU‐*rps*16, *trn*G‐UCC‐*trn*R‐UCU, *rpo*C2, *trn*S‐UGA, *trn*T‐UGU‐*trn*L‐UAA, *rps*19, *nad*F, *nad*F‐*rpl*32, *rpl*32‐*trn*L‐UAG, and *ycf*1 (Figure 7B).

These regions were identified within protein‐coding genes as well as intergenic regions. Similar to findings in other angiosperms, the IRs and protein‐coding segments displayed lower levels of divergence in comparison to single‐copy and non‐coding regions, as noted in prior studies (Wu et al. 2021; Zhang et al. 2022). Regions of divergence in plastid genomes were often utilized to distinguish closely related plant species (Dong et al. 2021; Zhang, Zhai, et al. 2025). Therefore, it was recommended to integrate several regions to improve the accuracy of the results. As a result, the 11 highly variable regions pinpointed in this investigation may function as potential DNA barcodes for *Artocarpus*.

## 5 Conclusions

This study offers the first comprehensive analysis of plastomes from 10 *Artocarpus* species in China, revealing insights into their structural evolution, genetic diversity, and phylogenetic relationships. Our results demonstrated that plastomes exhibited a conserved quadripartite architecture, with minor variations in length and GC content, and uniformly encode 132 genes, indicative of strong evolutionary constraints on core plastid functions. The identification of 73–95 SSRs and 51–81 dispersed repeats in each plastome offers valuable molecular markers for population genetics and species discrimination. The preference for A/U‐ending codons in codon usage is reflected in the AT‐rich plastid genomes, emphasizing the conservation of the translational machinery. The structure of the inverted repeats (IRs), notably the partial duplication of the *ycf*1 gene and the variable boundary distances of adjacent genes, highlights the plastic evolutionary processes that may drive genomic divergence. Selective pressure analyses indicated that purifying selection predominantly influenced most plastid genes, while 13 specific genes exhibited signatures of positive selection, suggesting adaptive evolution in reaction to environmental or functional pressures. Phylogenetic analyses of 42 Artocarpoideae plastomes strongly support the division of *Artocarpus* into two distinct clades, reinforcing established taxonomic relationships and offering a robust framework for resolving interspecific affinities within the genus. Additionally, nucleotide diversity region analyses revealed mutational hotspots, which offer promising targets for future phylogenetic and barcoding studies. Collectively, these results enhance our understanding of plastome evolution, genomic structure, genetic diversity, and phylogenetic relationships in *Artocarpus*. This work lays the foundation for elucidating the evolutionary history of *Artocarpus* and aids in developing tools for conservation, breeding, and species identification in this economically and ecologically significant genus.

## Author Contributions

**Ru‐Li Zhang:** conceptualization (lead), data curation (supporting), formal analysis (lead), writing – original draft (lead), writing – review and editing (lead). **Xian‐Huang Li:** visualization (lead), writing – original draft (supporting). **Shu‐Mei Nuo:** investigation (supporting). **Bi‐Lin Li:** investigation (supporting), methodology (supporting). **Ming‐Song Peng:** investigation (supporting), methodology (supporting). **Wei‐ying Li:** resources (supporting). **Yun Zhou:** investigation (supporting), methodology (supporting). **Dong Yan:** funding acquisition (lead), resources (lead), writing – review and editing (supporting). **Zhang‐Hong Dong:** data curation (lead), methodology (lead), project administration (supporting), writing – original draft (supporting), writing – review and editing (lead).

## Funding

This work was supported by Construction of the Near‐Site Conservation Base for Wild Plants with Extremely Small Populations in Nujiang, Gaoligong Mountain National Nature Reserve, Yunnan Province.

## Conflicts of Interest

The authors confirm no competing financial interests or personal relationships that could influence the work. All authors consented to manuscript submission.

## Supporting information


**Fig. S1.** Phylogenetic tree of 42 taxa from the subfamily Artocarpoideae, constructed using protein‐coding genes (PCGs) via Bayesian inference (BI) and maximum likelihood (ML). Values at each node indicate bootstrap support. Branch‐associated values represent BI posterior probabilities (PP) and ML bootstrap values (BS); asterisks (*) indicate bootstrap values/posterior probabilities of 100/1.00.

**Fig. S2.** Plastome alignments of ten *Artocarpus* species from China, with *A. heterophyllus* as the reference. *y*‐axis: sequence identity (50%–100%). Gray arrows mark gene positions and orientations. Red = conserved non‐coding sequences (CNSs); blue = exons of protein‐coding genes.

**Data S1:** Supplementary Tables.

## Data Availability

The annotated plastome was submitted to NCBI (https://www.ncbi.nlm.nih.gov/) under accession number PV740811.
