# Supplementary figures and images for: Comparative Plastome Analysis of Artocarpus Species in China: Insight Into Adaptive Evolution and Mutational Hotspot Regions
Source: Ecol Evol. 2026 Jan 2;16(1):e72881. doi: 10.1002/ece3.72881 (PMC12759183; doi:10.1002/ece3.72881)

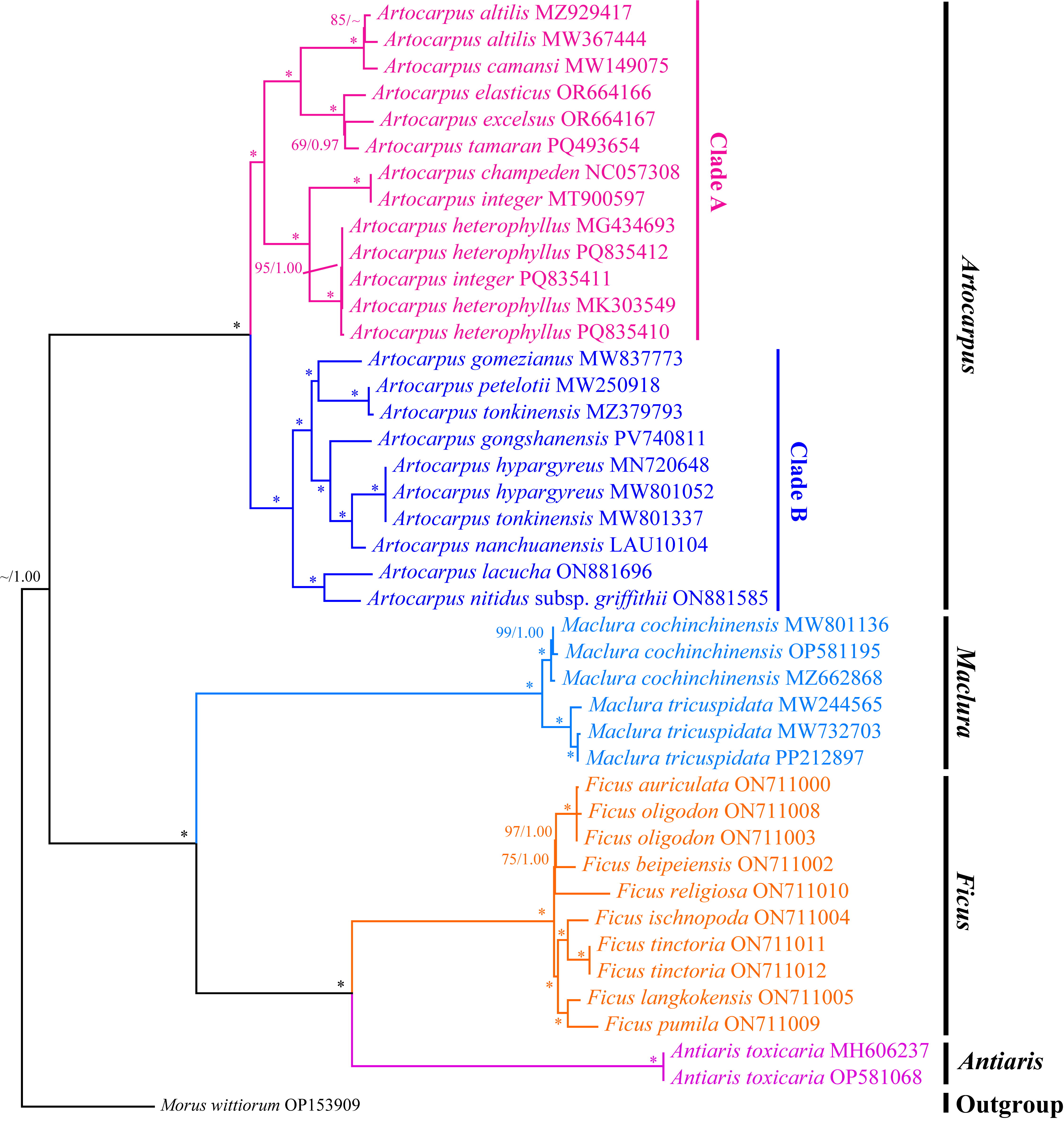

Supplement: Supplementary file 1 — Fig. S1. Phylogenetic tree of 42 taxa from the subfamily Artocarpoideae, constructed using protein‐coding genes (PCGs) via Bayesian inference (BI) and maximum likelihood (ML). Values at each node indicate bootstrap support. Branch‐associated values represent BI posterior probabilities (PP) and ML bootstrap values (BS); asterisks (*) indicate bootstrap values/posterior probabilities of 100/1.00. [file ECE3-16-e72881-s001.tif]

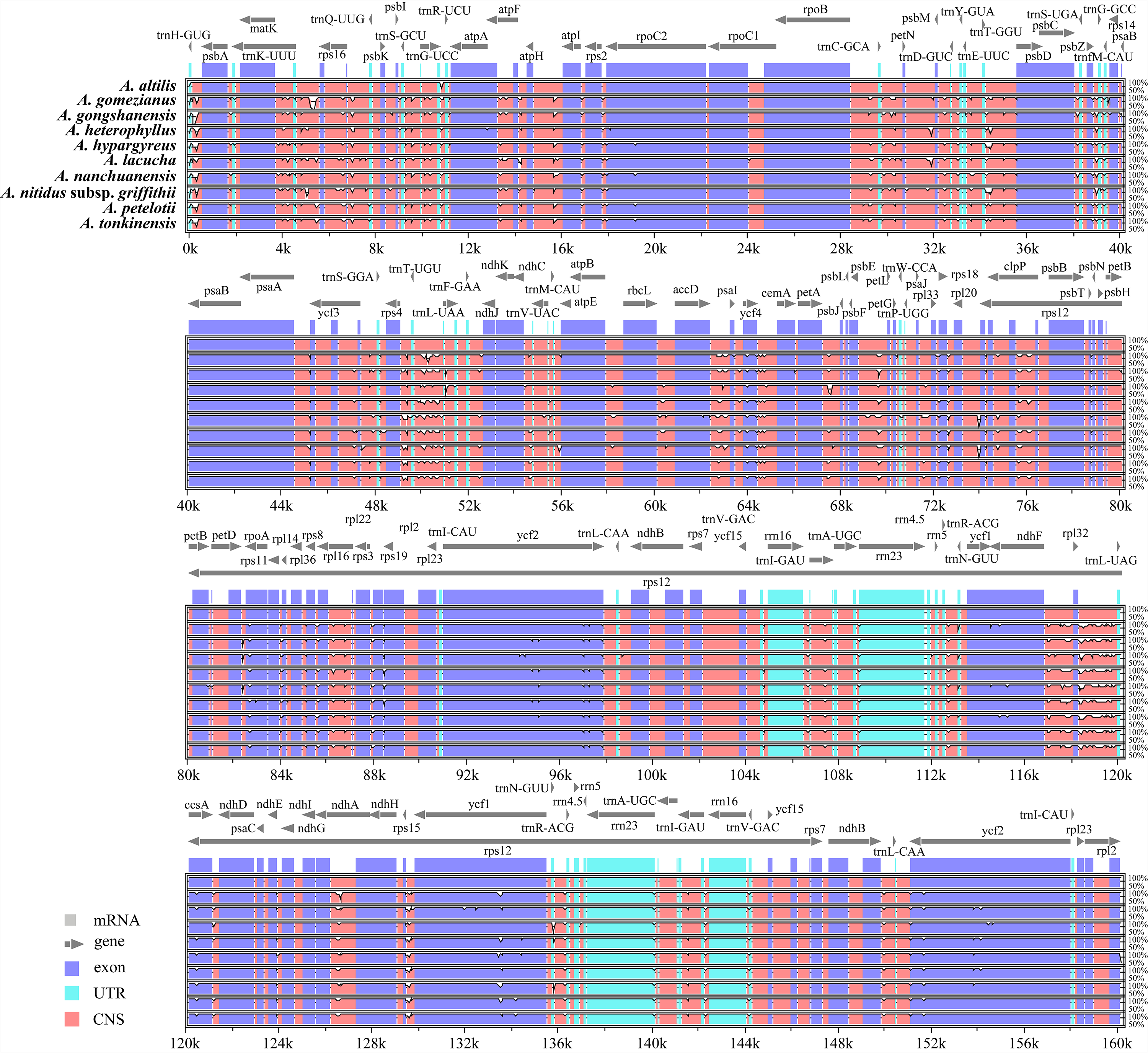

Supplement: Supplementary file 2 — Fig. S2. Plastome alignments of ten Artocarpus species from China, with A. heterophyllus as the reference. y‐axis: sequence identity (50%–100%). Gray arrows mark gene positions and orientations. Red = conserved non‐coding sequences (CNSs); blue = exons of protein‐coding genes. [file ECE3-16-e72881-s003.tif]
